# The complete mitochondrial genome of *Sphenomorphus indicus* (Reptilia: Scincidae)

**DOI:** 10.1080/23802359.2019.1644979

**Published:** 2019-07-23

**Authors:** Xin-Sheng Tang, Dian-Cheng Yang, Ya-Juan Lin, Liang-Liang Dai

**Affiliations:** College of Life and Environment Sciences, Huangshan University, Huangshan, P.R. China

**Keywords:** *Sphenomorphus indicus*, mitochondrial genome, phylogeny

## Abstract

The complete mitochondrial genome (mitogenome) sequence of *Sphenomorphus indicus* was sequenced and characterized by next-generation sequencing technology. The total length of mitogenome is 17,027 bp and contains 13 protein-coding genes, 22 tRNA genes, 2 ribosome RNA genes, and 2 non-coding regions (the control region and the putative L-strand replication origin). Most of the genes of *S. indicus* are encoded on the H-strand, except for the ND6 subunit gene and 8 tRNA genes which is distributed on the L-strand. Phylogenetic reconstruction suggested that *S. indicus* is the sister group of the *S*. *incognitus* within the genus *Sphenomorphus*. The complete mitochondrial genome sequence presented here will be useful to study the evolutionary relationships and genetic diversity of *S. indicus*.

The genus Sphenomorphus Fitzinger, 1843, is one of the most diverse groups of the family Scincidae with about 140 recognized species (Nguyen et al. 2011; Roy et al. 2013; Uetz et al. 2019). Only one complete mitochondrial genome (*Sphenomorphus incognitus*) was reported about this genus (Tang et al. 2019). *Sphenomorphus indicus* is widely distributed in the center and south of China, India, Nepal, Bhutan, Myanmar, Thailand, Laos, Vietnam, Cambodia, and south to west of Malaysia (Zhao et al. 1999; Uetz et al. 2019). In this study, the mitogenome of *S. indicus* was determined and described, in order to obtain basic genetic information about this species.

The specimen of *S*. *indicus* was collected from Yixian County, Huangshan, Anhui, China, and was preserved and deposited in the Museum of Huangshan University (Voucher number: HUM2017088). The complete mitogenome sequence of *S*. *indicus* was sequenced by next-generation sequencing technology (Illumina Hiseq 2500) at Shanghai Majorbio Bio-pharm Technology Co., Ltd (Shanghai, China). Genome sequences were assembled by the software SOAPdenovo v2.04 (Luo et al. 2012).

The complete mitogenome of *S*. *indicus* (Genbank accession number MK450438) was sequenced to be 17,027 bp which consisted of 13 typical vertebrate protein-coding genes, 22 transfer RNA (tRNA) genes, 2 ribosomal RNA (rRNA) genes, and 1 D-loop. The overall base composition of the genome is as follows: A (30.6%), T (25.5%), C (28.8%), and G (15.1%), of which the percentage of A + T is 56.1%. Most of the *S. indicus* mitochondrial genes are encoded on the H-strand except for the ND6 gene and 8 tRNA genes, which are encoded on the L-strand. All protein-coding genes use ATG as a start codon, except for the COI gene, which initiates with GTG. Eight of the 13 protein-coding genes (*ND1, COI, ATPase 8, ATPase 6, ND4L, ND5, ND6,* and *Cytb*) end with complete stop codons (*TAA, AGA*, and *AGG*), and the other five genes end with T as the incomplete stop codons, which were presumably completed as TAA by post-transcriptional polyadenylation (Anderson et al. 1981). The 12S rRNA (938 bp) and 16S rRNA (1526 bp) are located between the tRNA-Phe and tRNA-Lue genes and separated by the tRNA-Val gene. The D-loop of the *S. indicus* mitogenome in size is 1650 bp and is located between the tRNA-Pro and tRNA-Phe genes.

To validate the newly determined sequence, the whole mitochondrial genome sequence of the *S. indicus* determined in this study and together with other nine closely related species from GeneBank to perform phylogenetic analysis. A maximum likelihood (ML) tree was constructed based on the dataset by the online tool RAxML (Kozlov et al. 2018). Phylogenetic analysis result suggested that *S. indicus* is the sister group of the *S*. *incognitus* within the genus *Sphenomorphus* (Figure 1). It indicated that our newly determined mitogenome sequence could meet the demands and explain some evolution issues.

**Figure 1.**
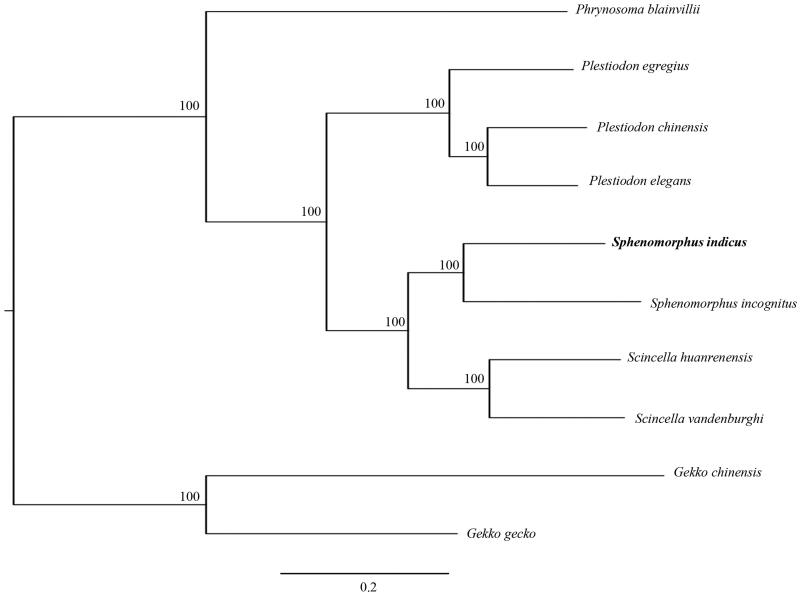
A maximum likelihood (ML) tree of the *Sphenomorphus indicus* in this study and other 9 closely related species was constructed based on the dataset of the whole mitochondrial genome by online tool RAxML. The numbers above the branch meant bootstrap value. Bold black branches highlighted the study species and corresponding phylogenetic classification. The analyzed species and corresponding NCBI accession numbers are as follows: *Phrynosoma blainvillii* (MG387969), *Plestiodon egregius* (AB016606), *P. chinensis* (KT279358), *P. elegans* (KJ643142), *Sphenomorphus indicus* (MK450438), *S. incognitus* (MH329292), *Scincella huanrenensis* (KU507306), *S. vandenburghi* (KU646826), *Gekko chinensis* (KP666135), *G. gecko* (AY282753).
